# PD104 Percutaneous Ethanol Injection In Thyroid Nodular Pathology And Metastatic Cervical Adenopathies: A Systematic Review, Meta-Analysis, And Economic Evaluation

**DOI:** 10.1017/S0266462324003490

**Published:** 2025-01-07

**Authors:** Beatriz León-salas, Aránzazu Hernández-Yumar, Diego Infante-Ventura, Aythami De Armas-Castellano, Yadira González-Hernández, Renata Linertová, Teresa Téllez-Santana, Pedro de Pablos-Velasco, Mar Trujillo-Martín

## Abstract

**Introduction:**

Percutaneous ethanol injection (PEI) is a valuable treatment for several health conditions. However, its beneficial and harmful effects in patients with thyroid nodular pathology and metastatic cervical adenopathies have not been assessed in a systematic review.

**Methods:**

A systematic review of available scientific literature on the safety, effectiveness, and cost effectiveness of PEI in thyroid nodular pathology and metastatic cervical adenopathies was performed according to Cochrane Collaboration methods and reported in accordance with the PRISMA statement. A cost-minimization analysis was carried out using a decision tree model. Assuming equal effectiveness between two minimally invasive techniques (PEI and radiofrequency ablation [RFA]), the model compared the costs of the alternatives with a horizon of six months from the perspective of the Spanish National Health System.

**Results:**

Three randomized controlled trials (n=157) evaluating PEI and RFA in patients diagnosed with benign thyroid nodules, 96 with predominantly cystic nodules and 61 with solid nodules, were identified. No evidence was found on other techniques or thyroid nodular pathology. No statistically significant differences were observed between PEI and RFA in proportion of volume reduction, symptom score, cosmetic score, therapeutic success, or major complications. No economic evaluations were identified. The cost-minimization analysis estimated the cost per patient of the PEI procedure to be EUR326, compared with EUR4,781 for RFA, with an incremental difference of −EUR4,455.

**Conclusions:**

There are no differences between PEI and RFA in terms of safety and effectiveness, but the economic evaluation determined that the former option is cheaper.

